# Establishment of a specimen panel for the decentralised technical evaluation of the sensitivity of 31 rapid diagnostic tests for SARS-CoV-2 antigen, Germany, September 2020 to April 2021

**DOI:** 10.2807/1560-7917.ES.2021.26.44.2100442

**Published:** 2021-11-04

**Authors:** Andreas Puyskens, Eva Krause, Janine Michel, C Micha Nübling, Heinrich Scheiblauer, Daniel Bourquain, Marica Grossegesse, Roman Valusenko, Victor M Corman, Christian Drosten, Katrin Zwirglmaier, Roman Wölfel, Constanze Lange, Jan Kramer, Johannes Friesen, Ralf Ignatius, Michael Müller, Jonas Schmidt-Chanasit, Petra Emmerich, Lars Schaade, Andreas Nitsche

**Affiliations:** 1 Robert Koch Institute, Highly Pathogenic Viruses, Centre for Biological Threats and Special Pathogens, WHO Reference Laboratory for SARS-CoV-2 and WHO Collaborating Centre for Emerging Infections and Biological Threats, Robert Koch Institute, Berlin, Germany; 2 Testing Laboratory for In-vitro Diagnostic Medical Devices, Paul-Ehrlich-Institute, Langen, Germany; 3 Charité – Universitätsmedizin Berlin, Institute of Virology and German Centre for Infection Research (DZIF), Associated Partner Site, Berlin, Germany; 4 Labor Berlin, Charité - Vivantes GmbH, Berlin, Germany; 5 Bundeswehr Institute of Microbiology and German Centre for Infection Research (DZIF), Partner Site Munich, Munich, Germany; 6 LADR Central Laboratory Dr. Kramer & Colleagues, Geesthacht, Germany; 7 MVZ Labor28 GmbH, Berlin, Germany; 8 Bernhard Nocht Institute for Tropical Medicine, Arbovirology Department, Hamburg, Germany; 9 Department of Tropical Medicine and Infectious Diseases, Center of Internal Medicine II, University of Rostock, Rostock, Germany

**Keywords:** antigen test, rapid diagnostic test (RDT), evaluation

## Abstract

**Introduction:**

The detection of SARS-CoV-2 with rapid diagnostic tests (RDT) has become an important tool to identify infected people and break infection chains. These RDT are usually based on antigen detection in a lateral flow approach.

**Aim:**

We aimed to establish a comprehensive specimen panel for the decentralised technical evaluation of SARS-CoV-2 antigen rapid diagnostic tests.

**Methods:**

While for PCR diagnostics the validation of a PCR assay is well established, there is no common validation strategy for antigen tests, including RDT. In this proof-of-principle study we present the establishment of a panel of 50 pooled clinical specimens that cover a SARS-CoV-2 concentration range from 1.1 × 10^9^ to 420 genome copies per mL of specimen. The panel was used to evaluate 31 RDT in up to six laboratories.

**Results:**

Our results show that there is considerable variation in the detection limits and the clinical sensitivity of different RDT. We show that the best RDT can be applied to reliably identify infectious individuals who present with SARS-CoV-2 loads down to 10^6^ genome copies per mL of specimen. For the identification of infected individuals with SARS-CoV-2 loads corresponding to less than 10^6^ genome copies per mL, only three RDT showed a clinical sensitivity of more than 60%.

**Conclusions:**

Sensitive RDT can be applied to identify infectious individuals with high viral loads but not to identify all infected individuals.

## Introduction

PCR-based diagnostics of severe acute respiratory syndrome coronavirus 2 (SARS-CoV-2) is a well-established method with numerous commercially available kits and in-house assays published over the last months [1]. Although it is beyond question that in particular real-time PCR provides an unrivalled degree of analytical sensitivity and reproducibility, there are some obvious limitations [2]. Above all, PCR-based diagnostics requires a functioning laboratory infrastructure and skilled personnel. The PCR reaction itself requires only around 2 h, including pre- and post-analytical steps; however, time to result in high-throughput mode is usually 24 h or more.

The need for faster and simpler approaches to diagnose a SARS-CoV-2 infection is therefore evident as well as the need for on-site tests and for diagnostics in regions with lower standards of laboratory infrastructure [3-5]. One promising technology to detect SARS-CoV-2-specific proteins in respiratory secretions are lateral flow immunoassays which operate within less than 30 min, so-called rapid diagnostic tests (RDT) [6]. The trade-off for a simple and quick diagnostic test is an often considerably lower analytical sensitivity and specificity compared with nucleic acid amplification techniques such as PCR [7,8].

Besides the traditional RDT whose read-out is performed visually, there are assays that utilise readers for the identification of positive signals. These readers can provide a better sensitivity, reproducibility and objectivity, in particular with fluorescence-based formats; however, the mobility of testing as well as the parallel testing of many specimens can be negatively affected.

Here we describe the decentralised evaluation of the sensitivity of 31 RDT using an identical panel of 50 clinical specimens analysed by up to six of seven participating German laboratories, namely those at the Robert Koch Institute, the Paul Ehrlich Institute, the Charité, as well as the Bundeswehr Institute of Microbiology, the LADR Central Laboratory Dr. Kramer & Colleagues, the MVZ Labor28 GmbH and at the Bernhard Nocht Institute for Tropical Medicine. With this approach, we generated at least two independent results per RDT and hence addressed inter-laboratory variations that have to be considered when RDT are performed in different locations by different persons.

## Methods

### Evaluation panel

To enable the systematic and comparable decentralised evaluation of numerous RDT, we compiled a panel of 50 samples by pooling a total of around 500 upper respiratory specimens from symptomatic patients, collected between March and September 2020 (Panel 1V1). For pooling, we mainly used dry swabs resuspended in phosphate-buffered saline (PBS) and a small number of swabs obtained in viral transport medium, resulting in a final concentration of viral transport medium of ≤ 20% in each pool, ≤ 10% in 38 pools and 0% in 25 pools.

Subsequently, up to 10 respiratory specimens obtained for routine diagnostics, with different virus loads as determined by real-time PCR, were pooled and diluted to a defined RNA load in a background of negative swabs in PBS. Pools were frozen at −80 °C. Real-time PCR was applied to determine the RNA load per pool [9]. In vitro RNA (provided by the World Health Organization (WHO)) as well as the quantitative reference material provided by INSTAND were used for quantification (https://www.instand-ev.de). Finally, the panel covered a range of SARS-CoV-2 RNA from 1.1 × 10^9^ genomes per mL down to 420 genomes per mL. When Panel 1V1 was used up, new pools were generated by diluting the same samples as for Panel 1V1 (except for four pools 1–4 that had to be constituted from new clinical specimens collected between October 2020 and January 2021), resulting in comparable virus loads as determined by real-time PCR, and that panel was labelled Panel 1V2. In addition, we compared Panel 1V1 with Panel 1V2 in RDT #3 and RDT #31 and the results were identical. Panel 1V1 and Panel 1V2 were later used to routinely evaluate the sensitivity of 122 RDT as described in the tandem publication by Scheiblauer et al. [10]. Here, we highlight the results of 31 of those RDT to give a more comprehensive insight into the performance of the evaluation panel. As negative control, we pooled respiratory specimens obtained by swabbing SARS-CoV-2-negative individuals.

Previous studies have revealed that a minimal RNA genome copy number of 10^6^ genome copies per mL of specimen represents the amount of infectious virus particles required for successful virus propagation in cell culture [9,11-14]. To correlate the pools to potential infectivity in a specimen, we propagated the pools with ≥ 10^6^ genome copies per mL, corresponding to a quantification cycle (Cq) value < 25, in cell culture. Confirmation of replication-competent SARS-CoV-2 was achieved by inoculation of VeroE6 cells with the respective pools. Pools containing infectious SARS-CoV-2 were subsequently titrated on VeroE6 cells. However, even if pools containing higher amounts of SARS-CoV-2 RNA generally showed higher titres than those with lower genome numbers, we observed no substantial correlation between the genome load and the titre (data not shown).

The specifications of the 50 pools are listed in Table 1; pools allowing SARS-CoV-2 propagation are marked in bold.

**Table 1:** Characteristics of the 50 pools of SARS-CoV-2 clinical specimens constituting Panel 1V1 and Panel 1V2, Germany, September 2020–April 2021

Panel 1V1			Panel 1V2		
Pool number	Cq/5 µL of RNA	RNA subjected to test	Pool number	Cq/5 µL of RNA	RNA subjected to test
1	**17.55**	1.1 × 10^7^	1	**17.31**	1.31 × 10^7^
2	**20.54**	1.4 × 10^6^	2	**19.08**	3.87 × 10^6^
3	20.38	1.6 × 10^6^	3	**19.62**	2.67 × 10^6^
4	**20.98**	1.0 × 10^6^	4	**20.61**	1.35 × 10^6^
5	**20.28**	1.7 × 10^6^	5	**20.60**	1.36 × 10^6^
6	**20.20**	1.8 × 10^6^	6	21.21	8.96 × 10^5^
7	**21.71**	6.4 × 10^5^	7	22.15	4.70 × 10^5^
8	21.95	5.4 × 10^5^	8	22.32	4.18 × 10^5^
9	22.14	4.7 × 10^5^	9	23.13	2.39 × 10^5^
10	22.88	2.8 × 10^5^	10	23.21	2.27 × 10^5^
11	22.34	4.1 × 10^5^	11	23.13	2.27 × 10^5^
12	**21.82**	5.9 × 10^5^	12	22.12	4.79 × 10^5^
13	23.32	2.1 × 10^5^	13	25.29	5.42 × 10^4^
14	24.28	1.1 × 10^5^	14	24.97	6.76 × 10^4^
15	**24.14**	1.2 × 10^5^	15	24.38	1.01 × 10^5^
16	22.55	3.6 × 10^5^	16	22.88	2.84 × 10^5^
17	24.00	1.3 × 10^5^	17	24.81	7.54 × 10^4^
18	25.30	5.4 × 10^4^	18	28.33	6.71 × 10^3^
19	25.50	4.7 × 10^4^	19	25.45	2.39 × 10^5^
20	26.27	2.8 × 10^4^	20	29.46	2.27 × 10^5^
21	25.54	4.6 × 10^4^	21	25.95	2.27 × 10^5^
22	25.87	3.7 × 10^4^	22	27.42	4.79 × 10^5^
23	**24.04**	1.3 × 10^5^	23	24.45	5.42 × 10^4^
24	25.24	5.6 × 10^4^	24	25.20	6.76 × 10^4^
25	29.70	2.6 × 10^3^	25	25.07	1.01 × 10^5^
26	25.47	4.8 × 10^4^	26	26.32	2.84 × 10^5^
27	25.14	6.0 × 10^4^	27	26.12	7.54 × 10^4^
28	27.14	1.5 × 10^4^	28	27.41	6.71 × 10^3^
29	27.15	1.5 × 10^4^	29	27.34	1.33 × 10^4^
30	28.86	4.7 × 10^3^	30	27.24	1.42 × 10^4^
31	25.27	5.5 × 10^4^	31	26.24	1.42 × 10^4^
32	26.44	2.5 × 10^4^	32	26.64	2.14 × 10^4^
33	28.96	4.4 × 10^3^	33	28.92	4.47 × 10^3^
34	27.89	9.1 × 10^3^	34	27.82	9.53 × 10^3^
35	27.04	1.6 × 10^4^	35	26.66	2.12 × 10^4^
36	28.13	7.7 × 10^3^	36	27.05	1.62 × 10^4^
37	30.54	1.5 × 10^3^	37	30.13	1.95 × 10^3^
38	28.14	7.6 × 10^3^	38	29.36	3.31 × 10^3^
39	29.76	2.5 × 10^3^	39	30.12	1.96 × 10^3^
40	27.65	1.1 × 10^4^	40	28.19	7.39 × 10^3^
41	30.13	1.9 × 10^3^	41	30.14	7.39 × 10^3^
42	28.43	6.2 × 10^3^	42	29.48	3.04 × 10^3^
43	31.05	1.0 × 10^3^	43	31.61	7.04 × 10^2^
44	29.24	3.6 × 10^3^	44	29.51	2.98 × 10^3^
45	30.10	2.0 × 10^3^	45	31.19	9.40 × 10^2^
46	31.54	7.4 × 10^2^	46	31.34	8.48 × 10^2^
47	35.19	6.0 × 10^1^	47	34.55	9.34 × 10^1^
48	32.06	5.2 × 10^2^	48	31.19	9.40 × 10^2^
49	35.22	5.9 × 10^1^	49	36.04	3.35 × 10^1^
50	36.36	2.7 × 10^1^	50	35.83	3.87 × 10^1^

Cq: quantification cycle; SARS-CoV-2: severe acute respiratory syndrome coronavirus 2.

Cq values in bold indicate that SARS-CoV-2 could be propagated in cell culture.

### Selection of rapid diagnostic tests

The RDT included in this study were selected at random according to availability at the time of the study (Table 2). No technical assumptions were made in the RDT selection process.

**Table 2:** SARS-CoV-2 rapid antigen tests evaluated in this study, Germany, September 2020–April 2021 (n = 31)

Number	Manufacturer/distributer (town, country)	Name	Number of evaluating laboratories	Target antigen
Classical point-of-care tests				
1	SD BIOSENSOR (Suwon-si, South Korea), distributed by Roche Diagnostics GmbH (Mannheim, Germany)	SARS-CoV-2 Rapid Antigen Test	6	N.s.
2	Abbott (Jena, Germany)	Panbio COVID-19 Ag Rapid Test Device (Nasopharyngeal)	5	N.s.
3	R-Biopharm AG (Darmstadt, Germany)	RIDA QUICK SARS-CoV-2 Antigen	3	N protein
4	servoprax GmbH (Wesel, Germany)	Cleartest	2	N.s.
5	Jiangsu Changfeng Medical Industry Co. (Touqiao Town, China), Ltd., distributed by nal von minden GmbH (Moers, Germany)	Dedicio COVID-19 Ag plus Test	3	N protein
6	nal von minden GmbH	NADAL COVID-19 Ag Test	3	N protein
7	RapiGEN Inc. (Gunpo-si, South Korea), distributed by Weko Pharma (Pellingen, Germany)	BIOCREDIT COVID-19 Ag	3	N.s.
8	SD BIOSENSOR	STANDARD Q COVID-19 Ag Test	2	N.s.
9	Biosynex Swiss SA (Freiburg, Switzerland)	BIOSYNEX COVID-19 Ag BSS	2	N protein
10	MEDsan GmbH (Hamburg, Germany)	MEDsan SARS-CoV-2 Antigen Rapid Test	2	N.s.
11	BIONOTE (Hwaseong-si, South Korea), distributed by concile (Freiburg, Germany)	NowCheck COVID-19 Ag Test	3	N.s.
12	Zhejiang Orient Gene Biotech Co., Ltd (Huzhou, China)	COVID-19 Rapid Ag Test Cassette	2	N protein
13	Fujirebio Inc. (Tokyo, Japan)	ESPLINE SARS-CoV-2	2	N.s.
14	Coris BioConcept (Gembloux, Belgium)	COVID-19 Ag Respi-Strip	2	N protein
15	Healgen Scientific LLC (Houston TX, US)	Healgen Coronavirus Ag Rapid Test Cassette (Swab)	2	N protein
16	Günter Keul GmbH (Steinfurt, Germany)	Keul-o-test	2	N.s.
17	Acro Biotech. Inc. (Rancho Cucamonga CA, US)	Acro Rapid Test COVID-19 Antigen Rapid Test (Nasopharyngeal Swab)	2	N protein
18	MEXACARE GmbH (Heidelberg, Germany)	COVID-19 Antigen Schnelltest (Nasen-Rachenabstrich)	2	N protein
19	Beijing Beier Bioengineering Co., Ltd. (Beijing, China)	COVID-19 Antigen Rapid Test Kit	2	N protein
20	möLaboratory GmbH (Langenfeld, Germany)	mö-screen Testkit Corona Antigen Nasenabstrich	2	N protein
21	Xiamen Biotime Biotechnology Co., Ltd. (Fujian, China), distributed by MEDICE Arzneimittel Pütter GmbH and Co. KG (Iserlohn, Germany)	Medicovid SARS-CoV-2 AG Antigen Schnelltest	2	N protein
22	Green Cross Medical Science Corp. (Eumseong-gun, South Korea)	GENEDIA COVID-19 Ag	2	N.s.
23	Guangdong Wesail Biotech Co., Ltd. (Dongguan, China), distributed by Bio-Gram Diagnostics GmbH (Ludwigshafen am Rhein, Germany)	COVID-19 Antigen Test Kit	2	N protein
24	Joinstar Biomedical Technology Co., Ltd. (Hangzhou, China), distributed by care impuls Vertriebs GmbH (Ried, Germany)	COVID-19 Antigen Schnelltest (Colloidal Gold)	2	N protein
25	Hangzhou Realy Tech Co. Ltd. (Hangzhou, China), distributed by TREKSTOR GmbH (Bensheim, Germany)	blnk COVID-19 Antigen Rapid Test (Nasopharyngeal Swab)	2	N.s.
26	Hangzhou Laihe Biotech Co., Ltd. (Hangzhou, China), distributed by Lissner Qi GmbH (Hamburg, Germany)	Novel Coronavirus (COVID-19) Antigen Test Kit	2	N protein
27	Koch Biotechnology (Beijing) Co., Ltd. (Beijing, China)	COVID-19 Antigen Rapid Test Strip	2	S protein
Rapid diagnostic tests requiring a read-out device				
28	SD Biosensor	STANDARD F COVID-19 Ag FIA	4	N protein
29	Quidel Corporation (San Diego CA, US)	Sofia SARS Antigen FIA	3	N protein
30	Schebo Biotech AG (Gießen, Germany)	ScheBo SARS-CoV-2 Quick Antigen	3	N protein
31	Beijing Wantai Biological Pharmacy (Beijing, China)	Wantai SARS-CoV-2 Antigen Rapid Test (FIA)	2	N protein

N: nucleocapsid; N.s.: not specified; S: spike.

### Rapid diagnostic test procedure

Except when RDT were evaluated without the included swabs, RDT were used according to the manufacturers' instructions. In brief, 50 µL of a pool were either directly added to the provided test buffer in volumes recommended by the manufacturer, or the swab included in the RDT kit was used to absorb the 50 µL and then subjected to the RDT procedure as recommended by the manufacturer. Results obtained by visual examination of the test device in different laboratories were categorised as 'positive' or 'negative' and subjected to statistical analysis by using the GraphPad Prism software as indicated in the Results section. Results were only accepted when the control band was positive, which was the case in more than 99% of the tested RDT.

### Ethical statement

The study obtained ethical approval by the Berliner Ärztekammer (Berlin Chamber of Physicians, Eth 20/40).

## Results

### Establishing the evaluation panel 

Before distributing the panel, we compared the detectability of specimens in two RDT (#2, #3) before and after freezing at −40 °C and did not observe any considerable differences in the virus concentration range close to the detection limit < 20,000 RNA copies per mL (data not shown). Furthermore, to test whether pooling and freezing had an impact on the detectability of specimens, we compared 40–44 fresh clinical specimens, representing a Cq value range from 20 to 35 (1.8 × 10^7^ to 7.0 × 10^3^) genome copies per mL, with 32 pools of Panel 1V1, covering a comparable Cq value range using 10 RDT (#2, #3, #7, #8, #10, #11, #14, #21, #28 and #31). In none of the RDT tested did we observe a considerable discrepancy between the detectability of fresh specimens and pools with a comparable Cq value (data summarised in Supplementary Figure S1).

Since some of the swabs had been transported to the laboratory in viral transport medium, the final concentration of viral transport medium in pools was ≤ 20% v/v for each pool, ≤ 10% v/v for 38 pools and 0% for 25 pools. Ten RDT were randomly selected to calculate whether viral transport medium had an impact on RDT sensitivity. Supplementary Figure S2 illustrates the results for one of the tested RDT; binary logistic regression revealed no difference in the detection probability of pools containing varying amounts of medium. Ultimately, we did not observe an influence of viral transport medium in the evaluation panel on RDT sensitivity.

### Analytical sensitivity of rapid diagnostic tests

**Figure 1:**
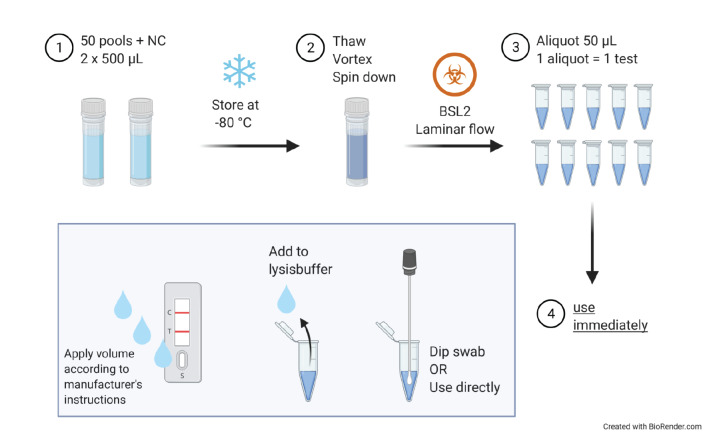
Recommendation for panel usage to guarantee maximum comparability between different laboratories and points in time of validation, evaluation of the sensitivity of 31 rapid detection tests for SARS-CoV-2 diagnostics, Germany, September 2020–April 2021

BSL: Biosafety level; NC: negative control.

All samples were stored at –40°C before shipment and were transferred to dry ice for shipping. The panels were shipped to the participating laboratories as two vials à 500 µL per sample. Upon arrival, samples were thawed, mixed and aliquoted in 50 µL aliquots to be used for testing. After thawing, aliquots were used immediately to allow maximum comparability between laboratories and test days. Figure 1 summarises the workflow as recommended to the participating laboratories. The results were assessed visually by experienced laboratory personnel.

**Figure 2:**
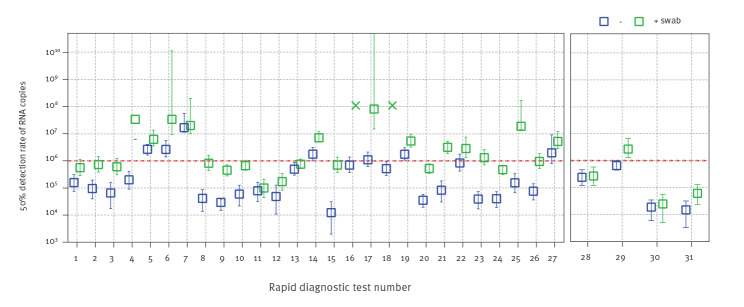
Analytical sensitivity of rapid diagnostic tests for SARS-CoV-2, expressed as 50% detection probability, Germany, September 2020–April 2021 (n = 31)

Figure 2 presents the sensitivity results for the 27 RDT that could be analysed visually (RDT #1–27) and the results for the four RDT that needed a device for read-out (RDT #28–31). Based on binary logistic regression of all results obtained from the different participating laboratories, the 50% probability to detect a certain genome load was calculated as a marker for the detection limit of an RDT. Seven of 31 RDT showed a 50% detection probability for genome loads higher than 10^6^ genome copies per mL, 24 had a 50% detection probability of less than 10^6^ genome copies per mL, while 15 had a 50% detection probability of less than 10^5^ genome copies per mL. The most sensitive RDT detected around 75,000 genome copies per mL with a probability of 90%, while the least sensitive RDT showed a 90% detection probability for 2.3 × 10^7^ genomes per mL.

Cq: quantification cycle; RDT: rapid diagnostic test.

RDT #28–31 require an additional device for reading out the result. Binary logistic regression was applied to calculate the analytical sensitivity from the 50 samples included in the panel. Open blue bars: virus-containing pools were directly subjected to the RDT without using a swab; open green squares: corresponding results when using a swab. Green crosses: tests that generated only one positive result for pool #1 (Cq = 17.55; 1.1 × 10^8^ RNA copies per mL), while the other 49 pools remained negative. Calculation of the 50% detection probability was not possible for these RDT.

Figure 2 also shows that using the RDT-specific swabs to absorb the pool material before testing can lead to a loss of analytical sensitivity at a factor of 10 to 50, although some of the RDT showed only small differences between direct application of the pool and swab usage. This indicates varying efficiency of the absorption and release characteristics for SARS-CoV-2 particles from the swabs.

### Clinical sensitivity of rapid diagnostic tests

To determine the clinical sensitivity of an RDT, results from different laboratories were merged and categorised according to the genome load, e.g. the Cq value obtained for each pool by real-time PCR [9]: Cq < 25 (10^6^ genome copies per mL), 25 ≤ Cq < 30 and Cq ≥ 30. Sensitivities of each RDT to identify pools correctly were calculated for each Cq category.

According to previous studies, Cq < 25 corresponds to an increased probability of a specimen to successfully propagate SARS-CoV-2 in cell culture [9,11,12]. This was confirmed for nine of 18 pools with Cq values < 25 for Panel 1V1 and five of 17 pools for Panel 1V2 (see bold entries in Table 1). Pools with a Cq ≥ 30 were highly unlikely to contain virus amounts high enough to grow in cell culture. Specimens with Cq values between 25 and 30 very rarely propagated virus in cell culture.

**Figure 3:**
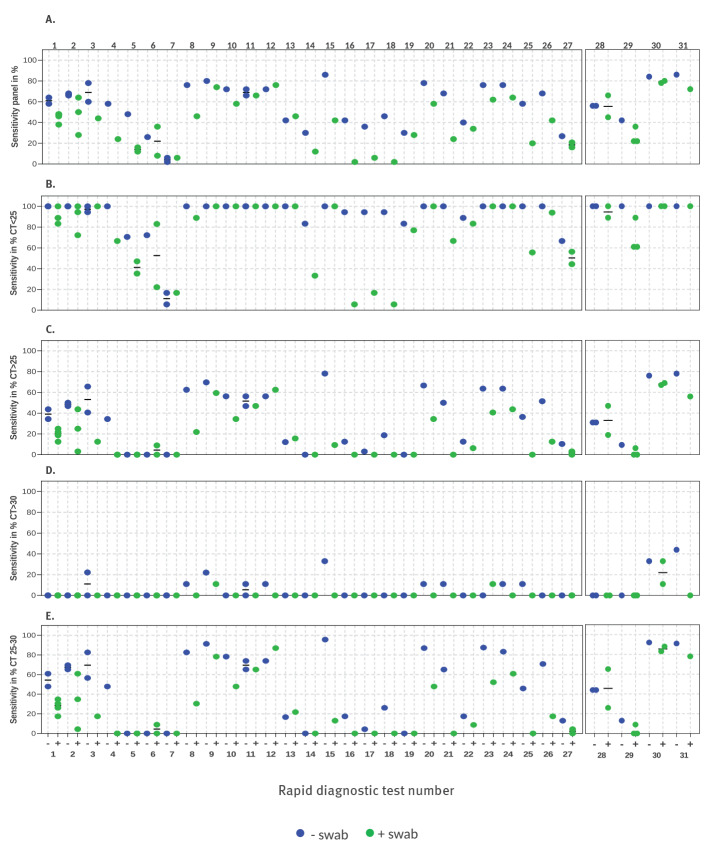
Clinical sensitivities of rapid diagnostic tests for SARS-CoV-2 as determined by two to six laboratories using 50 pools from evaluation Panels 1V1 and 1V2, Germany, September 2020–April 2021 (n = 31)

Figure 3 summarises the sensitivities for the 31 RDT. Figure 3A shows the sensitivity for all 50 SARS-CoV-2-positive pools of the panel. Results are further categorised according to the different virus loads represented by a pool. Figure 3B depicts pools with Cq < 25 (n = 18 Panel 1V1, n = 17 Panel 1V2, potentially infectious). Figure 3C shows pools with Cq ≥ 25 (n = 32 Panel 1V1, n = 33 Panel 1V2) and Figure 3D pools with a Cq value between 25 and 30 which is the range where the RDT showed considerable differences (n = 23 for both panels). The number of laboratories contributing to a result is presented in Table 2.

Cq: quantification cycle; RDT: rapid diagnostic test.

Blue circles: direct application of the pool to the test buffer; green circles: RDT-specific swab used. Each circle represents the results of one laboratory (symbols may cover each other). RDT #28–31 require an additional device for reading of result.

For virus loads higher than 10^6^ genome copies per mL (Cq < 25), the sensitivity of 26 of the 31 RDT was higher than 80%, indicating that these RDT would potentially identify infectious specimens with a probability of 80% (Figure 3A). For these RDT, the proficiency to detect those pools that contained culturable SARS-CoV-2 was even better, with values up to 100% (data not shown). For a virus load ≤ 10^6^ genome copies per mL (Cq ≥ 25), none of the evaluated tests could surpass a sensitivity of 80% (Figure 3B). In the Cq range between 25 and 30, 10 of 31 RDT reached a sensitivity of 80% and higher, and five further RDT showed sensitivities only slightly below 80% (Figure 3D). Finally, when the sensitivity to detect all 50 pools of the panel was determined, only four of 31 RDT passed a sensitivity of 80% or higher; two of those four RDT required a detection device for read-out. However, 10 of 31 RDT showed an overall sensitivity higher than 70% for the full panel (Figure 3A). A minimal detection rate of 75% for Cq < 25 was finally used as pass criterion in the comparative evaluation of 122 RDT as described in the tandem publication by Scheiblauer et al. [10]. Using a swab to transfer the volume of the pool material in the RDT test procedure led in the majority of evaluated RDT to reduced sensitivity. Hence, as described for the analytical sensitivity based on the RNA detection limit, clinical sensitivities were lower for most RDT when a swab was used.

Even if most of the RDT were analysed in two independent laboratories only, RDT #1–5 and #28–30 were evaluated by three to six laboratories with or without using swabs. As shown in Figures 2 and 3, there can be considerable variability for some tests, which is most probably due to the subjective interpretation of a positive test band; however, most results were very similar across laboratories and on different days.

## Discussion

RDT are promising tools in the diagnostic portfolio of tools for the identification of SARS-CoV-2-infected individuals [14-16]. Since these tests, unlike PCR, do not use amplification of their target molecule, their analytical sensitivity is usually limited. Hence, the evaluation of RDT plays a major role in defining the suitable scenarios for the use of RDT. In contrast to PCR, where the specimen can be inactivated, RDT should be evaluated with clinical material that contains native viruses to mirror the diagnostic application as authentically as possible. However, the systematic comparison of various RDT in different laboratories at different times requires larger sample volumes and good storage stability.

Multiple sampling of naso- and/or oropharyngeal swabs is hampered by reproducibility. Even sampling the same patient with several swabs consecutively is likely to result in different viral loads per swab, which has to be controlled by real-time PCR and changes the test procedure. Because most RDT protocols require the clinical specimen to be sampled with a swab from which virus has to be eluted in the system-specific buffer, one swab cannot be used more than once without changing the protocol. Therefore, the decentralised evaluation of various RDT in different laboratories is difficult. So far, clinical samples with semi-quantified SARS-CoV-2 concentrations or virus propagated in cell culture have been used to validate RDT [17-19]. This can be done for a limited number of tests in a short period of time but is not suitable when numerous RDT have to be compared regularly in different laboratories.

Therefore, we established an evaluation panel that was used to determine the analytical and clinical sensitivity of RDT providing comparable results. The main basis of each pool were dry swabs in PBS originally used for PCR diagnostics. Since some of the swabs had been transported to the laboratory in viral transport medium, the final concentration of viral transport medium differed between pools. Although we confirmed in only 10 of the evaluated RDT that the presence of viral transport medium did not influence RDT sensitivity, we believe that this low medium content in the evaluation panel did not influence the test sensitivity in general, since pools with medium percentages between 10% and 20% were distributed across the whole Panel 1V1.

We determined the SARS-CoV-2 genome load by real-time PCR in clinical specimens, and specimens with similar load were pooled, diluted in a background of negative swabs in PBS and the virus load quantified again. The established pools had a volume of 10 mL for both Panel 1V1 and 1V2 and covered a genome load from 1.1 × 10^9^ to 400 genomes per mL, which is the range of typical clinical specimens analysed in our laboratory. Even if the genome load does not reflect the number of virus particles directly, the RNA copy number was recently used to estimate the number of virus particles, reflecting the infectious potential of a specimen, and can correlate with the N protein concentration in clinical samples [20].

The fact that we used pools of up to 10 clinical specimens facilitates to some degree the compensation for potential variation between individual samples, for example varying ratios of genome copies vs number of viral particles or rather vs antigen concentration.

The pools of the panel showed results comparable to fresh clinical specimens with a similar SARS-CoV-2 genome load when selected RDT were used; freezing at −40 to −80 °C did not impact the detectability considerably. Nevertheless, as a trade-off for better reproducibility and comparability, the material used was not as fresh as in a clinical setting.

Usually, RDT use swabs that are subjected to the RDT-specific buffer before incubation of the test membrane. Our approach necessarily began with liquid specimens of 50 µL, which is intended for some RDT but not for all of them. However, with the intention to generate comparable and reproducible data, we accepted that some of the buffers were diluted by the fluid of the pools. Adding 50 µL of pools directly to three randomly selected RDT without application of additional test-specific buffers, we observed that detectability was not impaired in comparison to the regular protocol, at least not in these RDT.

The strategy of using liquid specimens comes with a further benefit, e.g. the option to cultivate the pools in cell culture, showing the infectivity of pools with a sufficient virus load. To our knowledge, this is one of the few studies that systematically evaluated several commercially available antigen RDT using standardised samples in comparison with real-time PCR as well as infectivity data from cell culture [21]. However, since all specimens included in a pool have been frozen and thawed at least once, the capability to grow in cell culture can be improved with fresh clinical specimens of a comparable virus amount [22].

Variability in the interpretation of RDT results observed in different laboratories has been described for diseases such as malaria and was significant for some RDT but, in general, highly comparable results were obtained [23]. Therefore, our results reflect the natural variance that can be expected when different users apply the RDT. It can be assumed that the interpretation of results is standardised better with RDT that require a device to read out the signal. Based on the number of RDT we have validated, we can confirm that read-out devices may help generate more reproducible results and reduce the inter-user variance.

The common RDT starts with the sampling that results in a swab containing material from the mucosa of the naso- or oropharynx. Then the swab is transferred into the RDT-specific buffer and subjected to the test membrane. Applying liquid evaluation specimens with known virus amounts does therefore not consider the impact of the swab on the result. The swab has to absorb liquids from the mucosa that potentially contain SARS-CoV-2 or can scrape off cellular material containing virus; probably it is a combination of both. Besides the problem that the swab does not absorb the specimen quantitatively, virus proteins can be retained by the swab and subsequently will not be subjected to the RDT, reducing the analytical sensitivity. While some of the RDT did not suffer considerably from using a swab before testing (#12, #13, #28 and #30), most of the tests lost sensitivity by a factor of 10 to 50. This does not mean that the respective test device is inferior but rather that the swab is not efficient in absorbing and releasing SARS-CoV-2 from a liquid specimen. However, speculating that these swabs will come with the same drawbacks when used in clinical sampling, the loss of sensitivity can also occur. In a patient carrying for example 10^6^ genome copies in the nasopharynx, with RNA load used as a surrogate for viral particles, the sampling on the mucosa bears a risk of a false negative result in the RDT because of considerable loss of antigen in the swab. Further investigations of the efficiency of virus absorption and release from a swab will help interpret the risk of false negatives by sampling. Besides, further specimen types such as saliva are under investigation for their use in RDT. Along these lines, we are currently further investigating the role of swab types on the uptake and release of virus material (study ongoing).

Finally, our study did not address the specificity of an RDT and cannot assess the risk of false positive results. At least all assessed RDT were negative for the negative control. However, recent studies show that high specificity is reached by most of the RDT evaluated [24]. In addition to SARS-CoV-2-positive specimens, the next version of our evaluation panel will also include SARS-CoV-2-negative specimens positive for other respiratory viruses.

### Conclusion

The sensitivity of the 31 RDT evaluated in this study varied extensively and depended largely on the virus load in the respective specimen. While four RDT showed a sensitivity of > 80% over the whole range of virus loads investigated, 26 RDT had a sensitivity of > 80% for potentially infectious specimens, indicating that sensitive RDT can be used to identify contagious individuals in various settings, but not to identify infected individuals with lower virus loads. Our results are in agreement with several other studies not using a standardised evaluation panel [25], indicating the applicability of the described panel for RDT evaluation. The minimal performance characteristics of an RDT have recently been discussed by the WHO to be at least 80% for symptomatic patients [15]. Considering that virus loads vary during the time course of infection in an individual and between individuals, the sensitivity of an RDT should rather be attributed to a certain virus load rather than the time after onset of symptoms or after a qualitative PCR result.

## Supplementary Data

149000 Supplement
