# Supplementary material for: Establishment of a specimen panel for the decentralised technical evaluation of the sensitivity of 31 rapid diagnostic tests for SARS-CoV-2 antigen, Germany, September 2020 to April 2021
Source: Euro Surveill. 2021 Nov 4;26(44):2100442. doi: 10.2807/1560-7917.ES.2021.26.44.2100442 (PMC8569922; doi:10.2807/1560-7917.ES.2021.26.44.2100442)
Supplement: Supplement [file 21-00442_NITSCHE_Supplement.pdf]

https://www.eurosurveillance.org/for-authors#Supplements:
This supplementary material is hosted by Eurosurveillance as supporting information alongside the article **Establishment of a specimen panel for the decentralized technical evaluation of the sensitivity of 31 rapid detection tests for SARS-CoV-2 diagnostics from September 2020 to April 2021 in Germany**, on behalf of the authors, who remain responsible for the accuracy and appropriateness of the content. The same standards for ethics, copyright, attributions and permissions as for the article apply. Supplements are not edited by Eurosurveillance and the journal is not responsible for the maintenance of any links or email addresses provided therein.

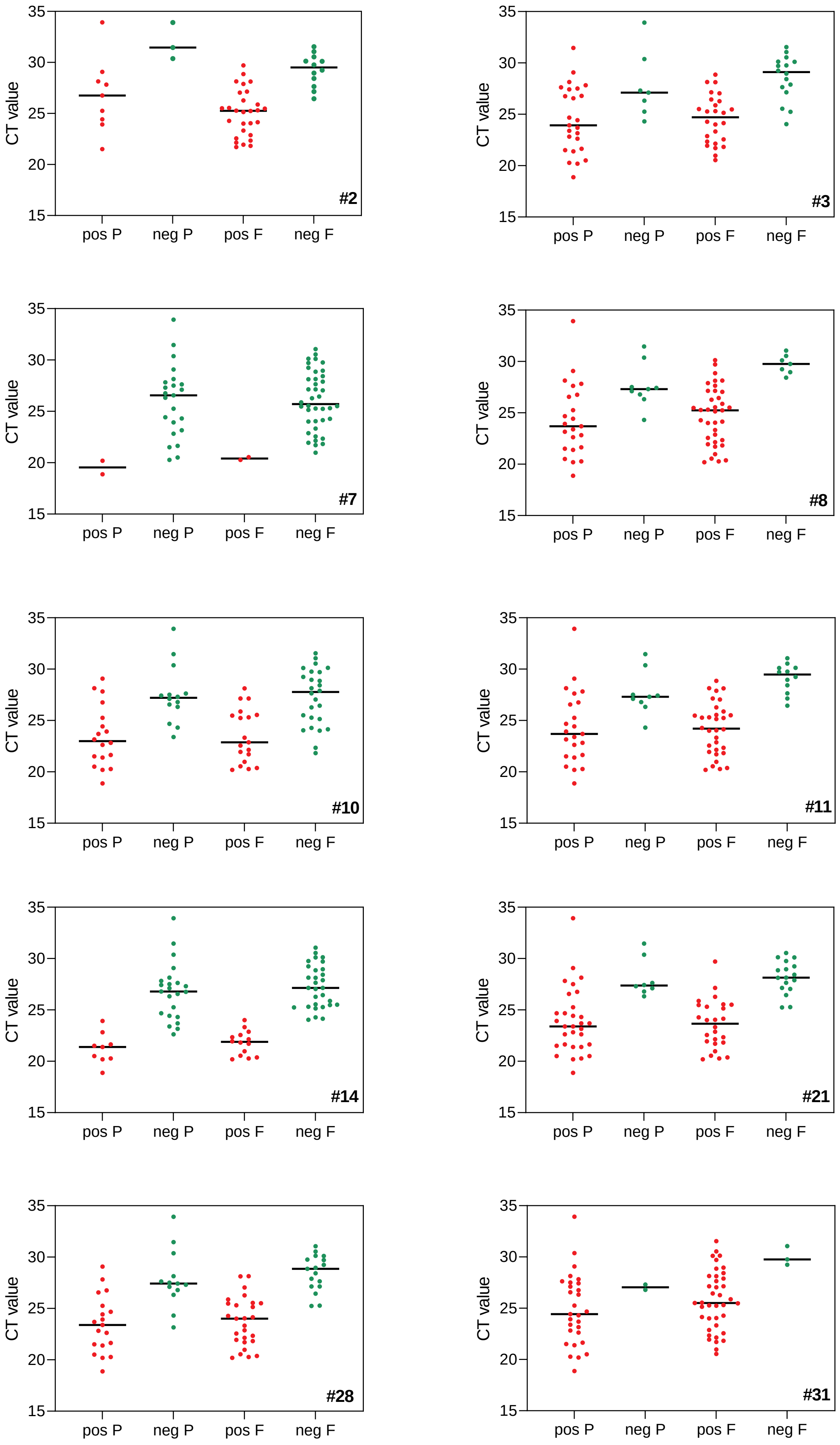
CT value
35
30
25
20
15
pos P
neg P
pos F
neg F
#2
#3
#7
#8
#10
#11
#14
#21
#28
#31

Supplementary figure S1: Comparison of 10 randomly selected RDTs with pools (P) **from Panel 1V1 (n≤32) and fresh (F) clinical specimens (n≤44), covering a genome** load from approximately 10^7 to $7x10^3$ genome copies per mL. None of the RDTs showed a significant difference in the detectability of pools in comparison to fresh specimens.

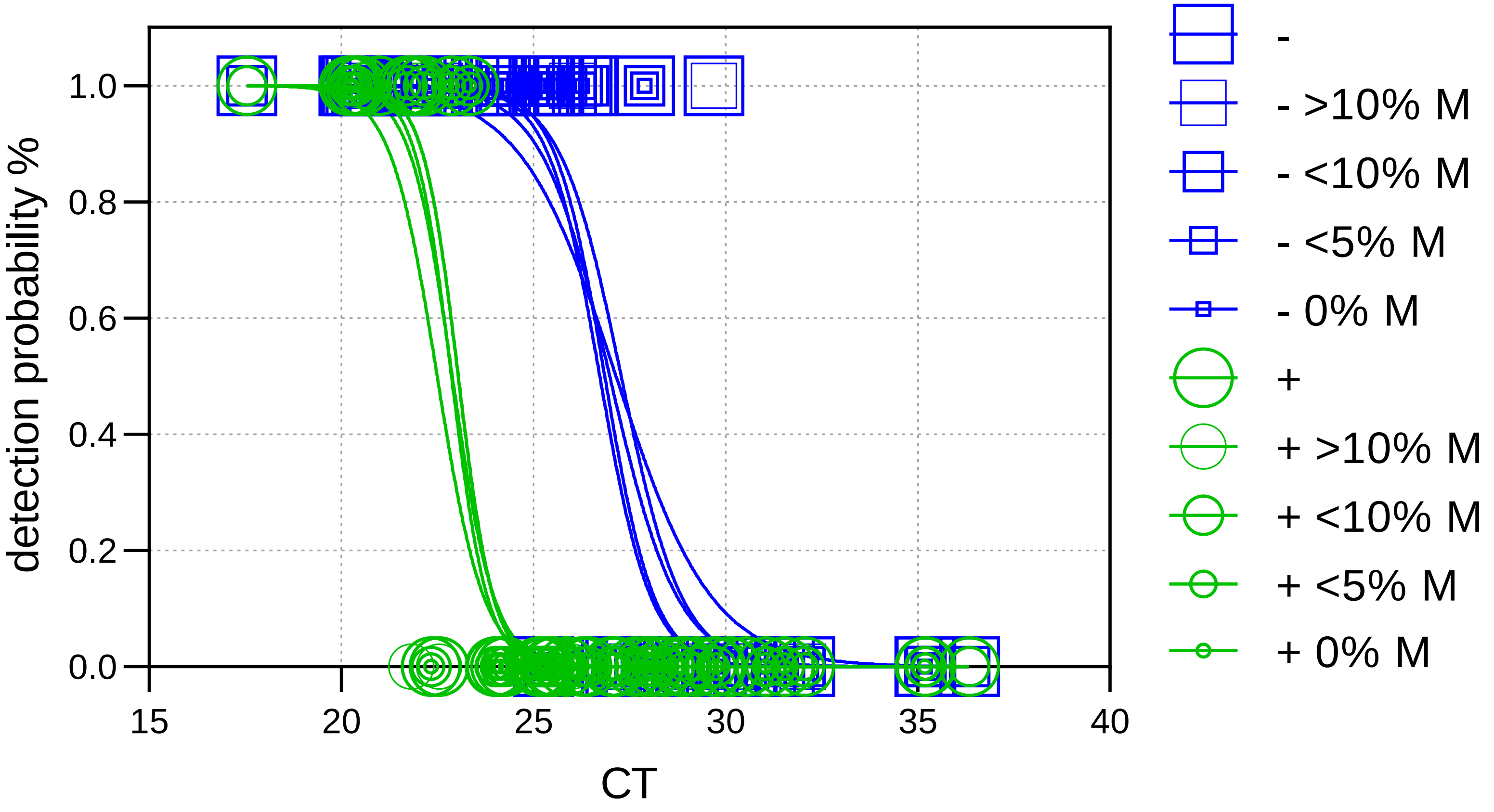

Supplementary figure S2: Impact of medium contents in the 50 pools included in Panel 1V1. Binary logistic regression was used to demonstrate that the amount of medium ranging from 0% to 20% has no impact on the detection limit of one randomly selected RDT.
